# AN INNOVATIVE APPROACH TO ENHANCING COPD CARE AND MANAGEMENT IN A RURAL NORTHERN COMMUNITY

**DOI:** 10.1093/geroni/igac059.2025

**Published:** 2022-12-20

**Authors:** Shannon Freeman, Anthon Meyer, Kelly Skinner, Laura Peach

**Affiliations:** University of Northern British Columbia, Prince George, British Columbia, Canada; Northern Health Authority, Prince George, British Columbia, Canada; University of Waterloo, Waterloo, Ontario, Canada; University of Waterloo, Waterloo, Ontario, Canada

## Abstract

**Background:**

COPD is the third leading cause of death worldwide. Rural communities often face challenges to provide high quality chronic disease care for aging populations. Despite these longstanding challenges, there was an intention to improve the care setting by developing and fostering a shared vision for quality care, as evidenced by enhancing COPD screening and care. To ensure consistent and longitudinal patient access to high quality of care as well and ongoing physician recruitment and retention a new rural program was developed. Objective-In this presentation we will describe a new rural community based COPD program from conceptualization and development through to current functioning highlighting areas of innovation. Methods-A process evaluation guided by Moore et al.’s framework to assess program implementation, mechanisms of impact, and context was conducted. Qualitative thematic analysis was undertaken of stakeholder interviews conducted in 2021 (n=11) and document review (n=60;~500 pages) of key clinic documents dated back to pre-program development.

**Results:**

We describe five phases of program development: Survive; Reorganize and Stabilize; Assess and Respond; Build and Refine; and Sustain and Share. Outreach and localizing resources improved access to the program. Acquiring secured physician compensation, capturing quality data, and improving patient and provider self-efficacy built the capacity of the system and stakeholders within it. Finally, relationships were forged through building an integrated facility, collaborative networking, and patient engagement. The key elements of program implementation were the resources required to ensure its operation, categorized as hardware, software, organizational, and human.

